# Single Incision Laparoscopic Appendectomy Using a New Multi-joint Articulating Instrument

**DOI:** 10.1007/s11605-021-05026-w

**Published:** 2021-05-19

**Authors:** Hyeong Yong Jin, Chul Seung Lee, Yoon Suk Lee

**Affiliations:** Department of Surgery, Division of Colorectal Surgery, Seoul St. Mary’s Hospital, College of Medicine, The Catholic University of Korea, Seoul, Republic of Korea

**Keywords:** Articulating instrument, ArtiSential^®^, Appendectomy, Single incision

## Background

Appendectomy is the most common emergent abdominal surgery worldwide, and almost 250,000 appendectomies were performed in the USA each year.^1^, ^2^ According to several previously published randomized controlled trials and meta-analyses reports, single-incision laparoscopic appendectomy (SILA) has potential advantages of shorter length of hospital stay and better cosmetic satisfaction than conventional three-port laparoscopic appendectomy.^3^, ^4^ However, SILA is still technically challenging because of internal collision between laparoscopic instruments. Due to these technical difficulties, SILA has disadvantages of longer operating times and higher conversion rates. As an alternative, new laparoscopic articulating instruments were developed.

## Purpose

Conventional straight-fixed laparoscopic instruments have disadvantages of reduced dexterity, limited freedom of movement, and uncomfortable ergonomics. Therefore, it was hard for surgeons to get an effective angle and make effective traction and counter-traction during laparoscopic surgery. To overcome these limitations, a surgical robot system, the da Vinci (Intuitive Surgical Inc., Sunnyvale, CA), was developed. Robot system provides high-definition three-dimensional vision with enhanced dexterity, multi-joint instruments, tremor reduction, and comfortable ergonomics. However, it has cost versus benefits issue. A new laparoscopic articulating instrument has multi-joint structure that are synchronized with the surgeon’s hand, wrist, and finger movement. With this structure, it can provide 360° of movement. Its multiple degrees of movement allows a wide range of surgical procedures like using a robotic arm. In addition, it has cost-effectiveness compared to robotic surgery. In this video, we intended to show how to overcome internal collision and make a good surgical view through a new laparoscopic articulating instrument during SILA.

## Materials and Methods

**Fig. 1:**
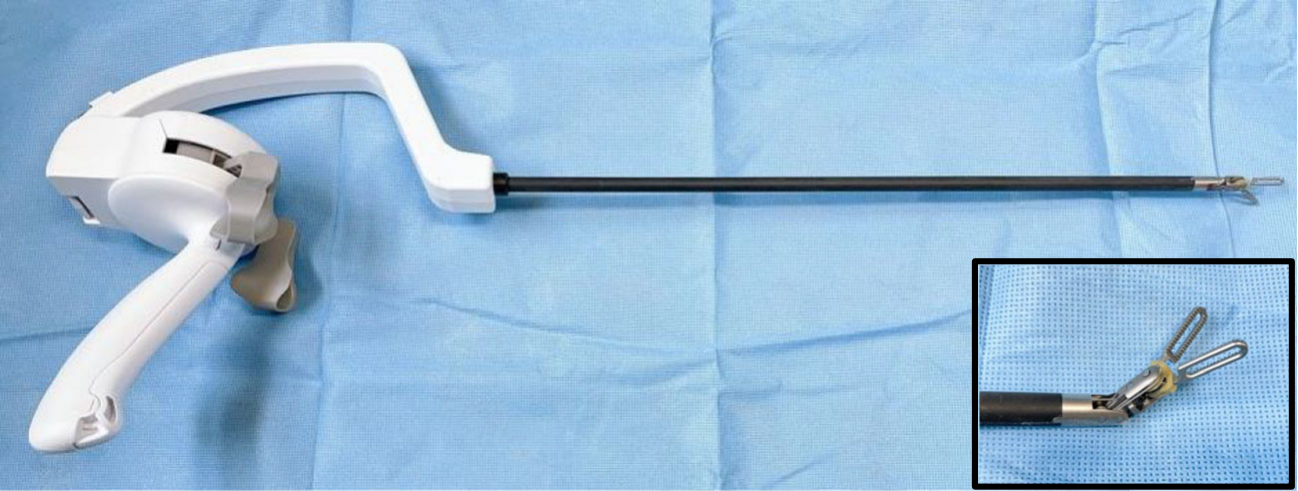
The laparoscopic multi-joint instrument (ArtiSential^®^)

We used the ArtiSential^®^ (LIVSMED Inc., Republic of Korea), a new laparoscopic articulating instrument which is registered as a class I medical device with the Korea Food and Drug Administration in 2019 (Fig. 1).^5^ Also, it achieved USFDA approval in June 2020. The instrument can be used with any 8 mm, or larger, sized trocar.

**Fig. 2:**
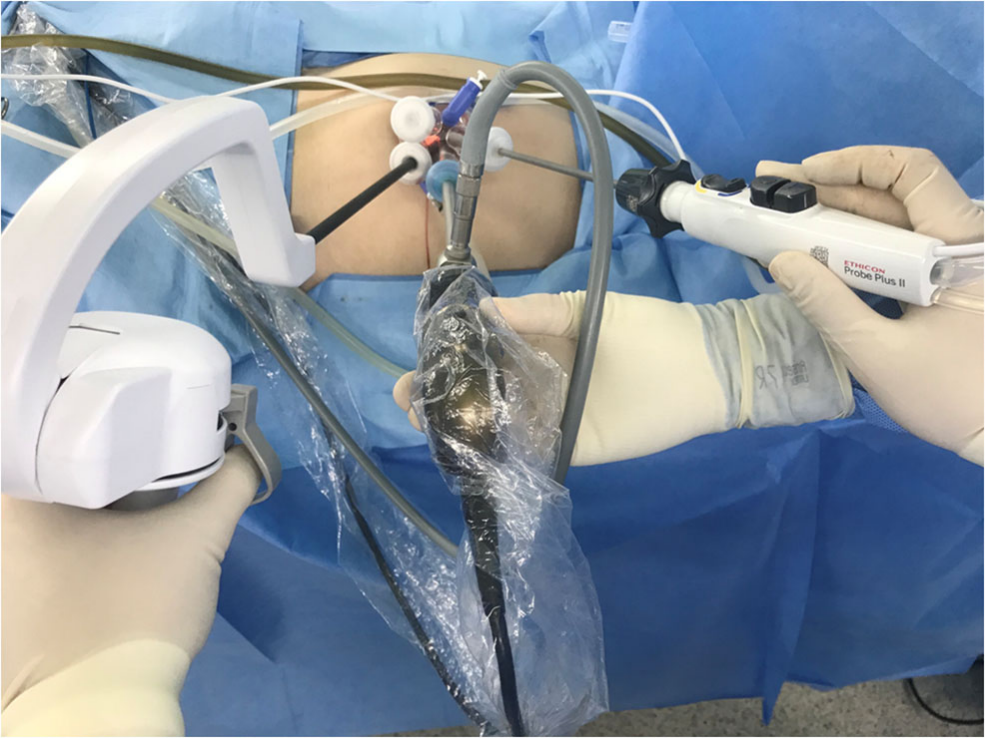
Port placement. The ArtiSential^®^ is placed on the left side, with surgeon’s non-dominant hand

In port placement, we applied the Glove port^®^ (Nelis Corp., Republic of Korea) (Fig. 2). It consists of three 5-mm-sized ports and one 12-mm-sized port. Therefore, it is possible to freely use a stapling device according to surgeon’s preference. The articulating instrument is placed on the left side, with surgeon’s non-dominant hand.

The articulating instrument is US$578, single port (Glove port^®^) is US$260, conventional laparoscopic instrument is US$210, and the conventional laparoscopic three ports are US$245 in Republic of Korea. There is no difference between the port prices, but the price of the instrument is expensive for the new instrument. However, it is very cheap compared to a robotic system.

This video article was approved by the Institutional Review Board of the Ethics Committee of the College of Medicine, The Catholic University of Korea (KC21ZASI0029).

## Results

In this video, we presented the steps of SILA using a new laparoscopic articulating instrument.

## Conclusions

Single-incision laparoscopic appendectomy using a new laparoscopic articulating instrument (ArtiSential^®^) is safe and technically feasible. Furthermore, it can be applied in various abdominal surgeries requiring wider range of movement.

## Supplementary Information

ESM 1 (MP4 217640 kb)
